# Temporal changes in self‐reported sleep quality, sleep duration and sleep medication use in relation to temporal changes in quality of life and work ability over a 1‐year period among Finnish municipal employees

**DOI:** 10.1111/jsr.13605

**Published:** 2022-04-15

**Authors:** Elina Bergman, Eliisa Löyttyniemi, Saana Myllyntausta, Päivi Rautava, Päivi E. Korhonen

**Affiliations:** ^1^ Department of General Practice, Institute of Clinical Medicine University of Turku and Turku University Hospital Turku Finland; ^2^ Department of Biostatistics University of Turku Turku Finland; ^3^ Department of Psychology and Speech‐Language Pathology University of Turku Turku Finland; ^4^ Department of Public Health University of Turku Turku Finland; ^5^ Turku University Hospital, Clinical Research Centre Turku Finland

**Keywords:** ability to work, occupational health, quality of sleep, well‐being

## Abstract

In this prospective follow‐up study, we aimed to examine whether changes in self‐reported sleep quality, sleep duration, and sleep medication use are temporally associated with changes in quality of life and work ability in municipal employees when several confounding factors are considered. The study was conducted in Finland among 637 municipal employees (88% women, mean [SD] age 48 [10] years) in 2014 and 2015. Information about the participants was collected by self‐administered questionnaire and from medical history. Predicting variables were changes in self‐reported sleep quality, sleep duration, and sleep medication use. Outcome variables were changes in the EUROHIS‐QOL eight‐item index and the Work Ability Score. Improved or unchanged sleep quality compared to worse sleep quality were associated with a preferable change in quality of life (both *p* < 0.001). No change in sleep duration compared to a decrease and no change in sleep medication use compared to increased use were also associated with favourable changes in quality of life. Increased use of sleep medication was associated with a decline in work ability, and the change in Work Ability Score also differed significantly between improved and worsened sleep quality. In this study, changes in sleep were widely associated with changes in quality of life and work ability of municipal employees. Programmes aiming for better sleep health would probably be beneficial both from a health‐oriented and an economical point of view. Special attention should be paid to employees with a need for sleep medication.

## 1 INTRODUCTION

Sleep is essential to our health, and people with sleep problems are at risk of developing several adverse health conditions. Depression (Baglioni et al., 2011), obesity (Itani et al., 2017; Jike et al., 2018; Madrid‐Valero et al., 2017; Patel & Hu, 2008), hypertension (Itani et al., 2017; Jike et al., 2018; Lo, Woo, Wong, & Tam, 2018; Wang et al., 2012), cardiovascular disease (Cappuccio et al., 2011; Itani et al., 2017; Kwok et al., 2018; Jike et al., 2018), and type 2 diabetes (Anothaisintawee et al., 2016; Cappuccio et al., 2010a; Itani et al., 2017; Jike et al., 2018) are more common in patients with sleep problems, and even all‐cause mortality has been linked with poor sleep quality and excessive short or long sleep duration (Cappuccio et al., 2010b; Itani et al., 2017; Kwok et al., 2018; Jike  et al.,^2018^). Besides affecting our physical and mental health, sleep issues also affect our quality of life (QoL) and ability to work. Excessive short or long sleep duration, poor sleep quality and chronic use of sleep medications have been linked with poorer outcomes in QoL (Bergman et al., 2020; LeBlanc et al., 2007; Liu et al., 2018; Marques et al., 2017; Tang et al., 2017) as well as work ability, sickness absence rate, and work productivity (Bergman et al., 2020; Ishibashi & Shimura, 2020; Lallukka et al., 2014, 2016; Lian et al., 2015). These findings make sleep a meaningful target for interventions especially if improvement in sleep parameters were associated with favourable changes in health and well‐being.

However, data about the associations of changes in sleep in relation to QoL are scarce, and to our knowledge, there is no information available in relation to work ability. In a study from the UK Household Longitudinal Survey (Tang et al., 2017) a positive change in sleep quality, increase in sleep quantity, and decrease in sleep medication use were all temporally associated with better subsequent health and well‐being, measured with the General Health Questionnaire (GHQ‐12) and the 12‐item Short‐Form Health Survey (SF‐12). However, the effects of chronic illnesses on the results were not considered in that paper. The presence of chronic illnesses has a negative effect on QoL (Schmidt et al., 2006) and work ability (Jędryka‐Góral et al., 2015; Shiri et al., 2013) according to several studies. Thus, a setting where chronic illnesses are not considered, ignore the possibility that chronic illnesses may be a moderator of the association between sleep and well‐being. In our own previous cross‐sectional works on public sector employees, we have shown that self‐reported sleep quality is significantly associated with QoL and work ability among the public sector active work force. The associations were detected in multivariable models with several predicting variables that could be associating with the outcomes (Bergman et al., 2019, 2020). These findings made us interested in examining the association of sleep variables with QoL and work ability in a more detailed manner and with a prospective study design.

The present study was based on the findings in previous literature as well as in our own previous works, and it was designed to address the gaps in previous research concerning the associations of changes in sleep in relation to QoL and work ability. This study aimed to examine whether changes in three relevant sleep parameters (i.e., self‐reported sleep quality, sleep duration, and sleep medication use) are temporally associated with changes in QoL (measured with the EUROHIS‐QOL eight‐item index) and work ability (measured with the Work Ability Score [WAS]) in municipal employees when several confounding factors, including chronic illnesses, are considered.

## 2 METHODS

### 2.1 Participants and study design

This prospective follow‐up study was part of the PORTAAT (PORi To Aid Against Threats) study conducted among employees of the city of Pori (83,497 inhabitants in 2014) in South‐Western Finland in 2014 and 2015. The participating work units were selected by the chief of the Welfare Unit of Pori. Invitations to participate and information of the study were sent to employees via email by the managers of the selected 10 work units (total number of employees 2,570). The employees willing to participate contacted the study contact person at their work unit, who then sent their contact information to the study nurse. There were no exclusion criteria. A total of 836 employees (104 males, 732 females) participated in the study in 2014. The response rate in 2014 was 32.5%. Complete information about data collection from that year has been described earlier (Veromaa et al., 2017). All the initial respondents were invited to the second part of the study in 2015, and 710 (85%) of them (79 males, 631 females) attended. The participants’ occupations included librarians, museum employees, janitors, information technology workers, social workers, nurses, physicians, administrative officials, and general office staff. The mean age and gender ratios of the study participants were comparable to the entire personnel of the city of Pori (2014). The gender distribution also resembles the distribution among employees of the Finnish public sector in general (Kouvonen et al., 2007).

For the analyses of the present study, 637 participants were included in the study population. The inclusion criteria were complete information about age, gender, educational level, and body mass index (BMI) at the first measurement point in 2014 and EUROHIS QOL eight‐item index, WAS, and sleep duration at both measurement points. Participants, who were pregnant at either time point (seven), were excluded. There were 126 participants who were excluded because they did not attend the follow‐up visit and additional 73 participants who were excluded due to missing information or pregnancy. The participants excluded from the analyses did not vary significantly from the study cohort in terms of gender, BMI, disease burden, sleep duration, sleep quality, or QoL. However, they were slightly older and less educated. They also used more sleep medications at the follow‐up visit, and their work ability had developed more unfavourably during the follow‐up period compared to the study cohort (Table S1).

All procedures performed in studies involving human participants were in accordance with the ethical standards of the institutional and/or national research committee and with the 1964 Helsinki Declaration and its later amendments or comparable ethical standards. The study protocol and consent forms were reviewed and approved by the Ethics Committee of the Hospital District of Southwest Finland (ETMK 43/180/2013). Written informed consent was obtained from all individual participants included in the study.

### 2.2 Assessment of QoL

The QoL was assessed with the EUROHIS‐QOL eight‐item index (Power, 2003). This is a shortened version of the WHOQOL‐BREF scale, a widely used instrument for the assessment of general QoL (The WHOQOL Group, 1998). The domains in both questionnaires are the general, physical, psychological, social, and environmental aspects of QoL. The EUROHIS‐QOL instrument has been validated in several European countries (Schmidt et al., 2006). The participants of the present study answered the questions at home before the study visits. Every question was scored from 1 (“very poor”) to 5 (“very good”). All scores were then added together and divided by 8 (*the sum of the questions*) to obtain the EUROHIS‐QOL mean score (Schmidt et al., 2006).

### 2.3 Assessment of work ability

Work ability was assessed with the question “What is your current work ability compared to your lifetime best?.” This is the first item of the widely used Work Ability Index (WAI; Ilmarinen, 2007) referred to as the WAS. It has a 0–10 response scale, where 0 stands for “completely unable to work” and 10 stands for “work ability at its best.” With the WAS, work ability is considered poor for scores of 0–5, moderate for scores of 6–7, good for scores of 8–9, and excellent for a score of 10 points, based on the same values that have been used in the WAI (Gould et al., 2008).

### 2.4 Assessment of sleep‐related measures

Self‐reported sleep quality was assessed with the question “During the past month, how would you rate your sleep quality overall?” (“very good”, “good”, “poor”, or “very poor”). Sleep duration was measured with the question “During the past month, how many hours of sleep did you normally get at night?.” The participants were asked to answer the question in an open text field, and during the data entry, the answers were rounded to the nearest half an hour. Similar rounding of self‐reported sleep duration has also been used in previous literature (Myllyntausta et al., 2017). Use of sleep medication was assessed with the question “During the past month, how often have you taken medicine (prescribed or ‘over the counter’) to help you sleep?” (“not during the past month”, “less than once a week”, “once or twice a week”, or “three or more times a week”). All these questions are items from the well‐validated Pittsburgh Sleep Quality Index (PSQI; Buysse et al., 1989).

Three response categories were derived for all three sleep parameters to describe change in these variables between the two measurement points. This approach was chosen because we wanted to explore especially the association of changes in sleep parameters with changes in outcome variables, regardless of the baseline results of the sleep measurements. For both sleep duration and use of sleep medication, the three categories were “increased”, “no change” and “decreased”; for sleep quality, the categories were “improved”, “no change” and “worse.” Any difference in sleep quality and sleep medication use and a difference of half an hour in sleep duration between the measurement points were considered as changes in the analyses.

### 2.5 Assessment of covariates

Basic measurements were performed at baseline in 2014. Height and weight were measured by a study nurse with subjects in standing position without shoes and outer garments. Weight was measured to the nearest 0.1 kg with calibrated scales and height to the nearest 0.5 cm with a wall‐mounted stadiometer. The BMI was calculated as weight (kg) divided by the square of height (m^2^) and categorised into two classes (<30 and ≥30 kg/m^2^) for the analyses. Age was categorised into three classes (<45, 45–55 and >55 years). Information concerning diseases diagnosed by a physician and vocational education level (“vocational school”, “college‐level education”, or “university‐level education”) was gathered using self‐administered questionnaires and medical records. Disease burden (“yes” versus “no”) was defined as having at least one chronic disease diagnosed by a physician.

### 2.6 Statistical analysis

Continuous variables are presented with means and with SDs or 95% confidence intervals (CIs) if the variable is normally distributed. Categorical variables are summarised with counts and percentages (%).

Models for multiway analysis of covariance were built to study factors that could be associated with EUROHIS‐QOL and WAS change. The outcome measures in the models were the mean changes of EUROHIS‐QOL and WAS between the years 2014 and 2015, which were calculated by subtracting the baseline measurement from the 2015 measurement. Change in sleep quality, sleep duration, and sleep medication use were all entered in different models (due to collinearity issues) separately for EUROHIS‐QOL change and WAS change. The results are reported as mean changes with 95% CIs, and with *F* values and degrees of freedom (DF). All the models were adjusted for age, gender, BMI, educational level, disease burden, and the studied outcome measure (EUROHIS‐QOL or WAS) level at baseline (2014). Multiple group comparisons between different classes of changes in sleep parameters in the adjusted models were also performed. Mean differences between the groups with 95% CIs are reported to indicate effect sizes.

All statistical tests were performed as two‐sided, with a significance level set at 0.05. For multiple group comparisons, Tukey's adjusted method was used. The analyses were performed using an SAS System version 9.4 for Windows (SAS Institute).

## 3 RESULTS

The study cohort consisted of 637 employees with a mean (SD) age of 48 (10) years at baseline, 88% of whom were female. The characteristics of the study cohort are presented in Table 1.

**TABLE 1:** Characteristics of the study population at baseline in 2014

Characteristic	*N* (%)
Gender	
Female	561 (88.1)
Male	76 (11.9)
Age (years)	
<45	212 (33.3)
45–55	235 (36.9)
>55	190 (29.8)
Body mass index (kg/m^2^)	
<30	498 (78.2)
≥30	139 (21.8)
Vocational education	
Vocational school	16 (2.5)
College level	322 (50.6)
University level	299 (46.9)
Disease burden^a^	
Yes	383 (60.1)
No	254 (39.9)

^a^ Disease burden was defined as having at least one chronic disease diagnosed by a physician.

At baseline, 72% of the participants had good or very good sleep quality, sleep duration was 7–8 h/night in 70% of the participants, and 85% did not use sleep medications. Sleep quality and sleep medication use had remained unchanged in most of the participants at the follow‐up visit, but changes in sleep duration were more common. However, most of the changes in sleep duration were minor, and ~90% of the participants reported either unchanged sleep duration or sleep duration that had changed ± 1 h at most. EUROHIS‐QOL and WAS mean scores had slightly improved during the follow‐up (Table 2). The division of the participants according to changes in sleep parameters presented in Table 2 was used in further analyses. Of note, the results in the groups of “no change” in all the three sleep parameters were on a favourable level already at the baseline: In the group of no change in sleep quality, the reported level of sleep quality was good or very good in 81% of the participants. In the group of no change in sleep duration, 72% of the participants slept 7–8 h/night, and in the group of no change in sleep medication use, only 7% of the participants had used sleep medications during the past month (data not shown).

**TABLE 2:** Sleep quality, sleep duration and sleep medication use, and EUROHIS‐QOL and Work Ability Score means in the study population in 2014 and 2015, and the change between the years

	Year 2014	Year 2015	Change between 2014 and 2015
	*N* (%) or mean (95% CI)	*N* (%) or mean (95% CI)	*N* (%) or mean (95% CI)
Sleep quality			
Very good	71 (11.2)	95 (15.0)	
Good	385 (60.9)	397 (62.5)	
Poor	161 (25.5)	130 (20.5)	
Very poor	15 (2.4)	13 (2.1)	
Sleep quality change			
Worse			87 (13.8)
No change			404 (64.1)
Improved			139 (22.1)
Sleep duration			
<7 h/night	168 (26.4)	216 (33.9)	
7–8 h/night	445 (69.9)	387 (60.8)	
>8 h/night	24 (3.8)	34 (5.3)	
Sleep duration change			
Decreased			196 (30.8)
No change			248 (38.9)
Increased			193 (30.3)
Sleep medication use			
No	539 (85.2)	547 (86.3)	
<1 night/week	43 (6.8)	44 (6.9)	
1–2 nights/week	23 (3.6)	14 (2.2)	
≥3 nights/week	28 (4.4)	29 (4.6)	
Sleep medication use change			
Increased			40 (6.4)
No change			543 (86.2)
Decreased			47 (7.5)
EUROHIS‐QOL, mean	3.94 (3.90–3.98)	4.08 (4.04–4.12)	0.14 (0.11–0.17)
WAS, mean	8.16 (8.06–8.26)	8.36 (8.26–8.46)	0.20 (0.11–0.29)

CI, confidence interval; EUROHIS‐QOL, EUROHIS‐QOL eight‐item index; WAS, Work Ability Score.

### 3.1 Sleep and EUROHIS‐QOL change

Mean changes in EUROHIS‐QOL score between the years 2014 and 2015 (non‐adjusted and adjusted) are presented in Table 3. Changes in self‐reported sleep quality, sleep duration and sleep medication use were statistically significantly associated with changes in EUROHIS‐QOL scores in multivariable models adjusted for age, gender, BMI, educational level, disease burden and EUROHIS‐QOL level at the first measurement point.

**TABLE 3:** Changes is EUROHIS‐QOL eight‐item index according to changes in sleep parameters

Quality of life (EUROHIS‐QOL eight‐item index)							
	Mean change (95% CI)	Mean change adjusted estimate (95% CI)	*F* value (DF)	*p*	Group comparisons		
						Mean difference adjusted estimate (95% CI)	*p*
Sleep quality							
Worse	−0.02 (−0.10 to 0.07)	−0.05 (−0.15 to 0.05)	9.9 (2)	<0.0001	Worse vs. no change	−0.15 (−0.25 to −0.06)	0.0005
No change	0.14 (0.11–0.17)	0.10 (0.03 to 0.18)			Worse vs. improved	−0.21 (−0.32 to −0.10)	<0.0001
Improved	0.25 (0.18–0.32)	0.16 (0.07 to 0.24)			No change vs. improved	−0.06 (−0.14 to −0.03)	0.25
Sleep duration							
Decreased	0.08 (0.03–0.14)	0.05 (−0.03 to 0.13)	3.5 (2)	0.031	Decrease vs. no change	−0.08 (−0.16 to −0.00)	0.042
No change	0.18 (0.13–0.23)	0.13 (0.05 to 0.21)			Decrease vs. increase	−0.08 (−0.16 to 0.01)	0.075
Increased	0.15 (0.10–0.21)	0.13 (0.05 to 0.21)			No change vs. increase	0.00 (−0.07 to 0.08)	0.99
Sleep medication use							
Increased	0.08 (−0.07 to 0.22)	−0.06 (−0.19 to 0.07)	4.9 (2)	0.0076	Increase vs. no change	−0.17 (−0.31 to −0.04)	0.0073
No change	0.14 (0.11–0.18)	0.12 (0.04 to 0.19)			Increase vs. decrease	−0.12 (−0.29 to 0.06)	0.27
Decreased	0.17 (0.05–0.28)	0.06 (−0.07 to 0.18)			No change vs. decrease	0.06 (−0.07 to 0.18)	0.52

CI, confidence interval; DF, degrees of freedom; EUROHIS‐QOL, EUROHIS‐QOL eight‐item index.

*Note*: Mean changes (non‐adjusted and adjusted) with 95% CIs in EUROHIS‐QOL years 2014–2015 according to changes in sleep parameters. Adjusted mean values, *F* values, DF and *p* values are from three different models for multiway analysis of covariance to explain factors affecting the change in quality of life. Change in sleep quality, sleep duration and sleep medication use were all entered in different models with EUROHIS‐QOL change. All models were adjusted for age, gender, body mass index, educational level, disease burden, and EUROHIS‐QOL level at baseline. Group comparisons between different classes of sleep parameters are also presented. Mean differences in EUROHIS‐QOL between groups are presented with 95% CI. The *p* values and 95% CIs for group comparisons are adjusted with Tukey's method.

In pairwise comparisons, improved and unchanged sleep quality compared to worse sleep quality were associated with a preferable change in EUROHIS‐QOL. We also performed some additional exploratory analyses (data not shown), where the group of unchanged sleep quality was divided into continuously good or very good (81%) and continuously poor or very poor (19%) sleep quality. These analyses showed, that the EUROHIS‐QOL had improved significantly in the group of continuously good sleep quality during the follow‐up, but no change had occurred in the group of continuously poor sleep quality. According to this, the good results in the group of unchanged sleep quality are based on the results of the participants, whose sleep quality was continuously good or very good.

No change in sleep duration, compared to a decrease, was associated with a favourable change in EUROHIS‐QOL. No statistically significant differences were detected in EUROHIS‐QOL change between those whose sleep duration had changed >1 h compared to those with a smaller change of the same direction either in decreased or in increased sleep duration (data not shown). No change in sleep medication use compared to increased use was also associated with a favourable change in EUROHIS‐QOL (Table 3).

### 3.2 Sleep and WAS change

Mean changes in WAS between the years 2014 and 2015 (non‐adjusted and adjusted) are presented in Table 4. Changes in sleep quality and sleep medication use were statistically significantly associated with changes in WAS in multivariable adjusted models. Increased use of sleep medication was associated with a decline in WAS, and in pairwise comparisons, this result was statistically significantly different compared to the group with no change in sleep medication use. The change in WAS differed statistically significantly also between improved and worsened sleep quality. However, the change in WAS per se was not statistically significant in any of the groups of sleep quality during the follow‐up period (Table 4).

**TABLE 4:** Changes in Work Ability Score (WAS) according to changes in sleep parameters

Work ability (WAS)							
	Mean change (95% CI)	Mean change adjusted estimate (95% CI)	*F* value (DF)	*p*	Group comparisons		
						Mean difference adjusted estimate (95%CI)	*p*
Sleep quality							
Worse	−0.18 (−0.43 to 0.07)	−0.20 (−0.51 to 0.10)	3.8 (2)	0.022	Worse vs. no change	−0.25 (−0.54 to 0.04)	0.11
No change	0.18 (0.07 to 0.29)	0.05 (−0.18 to 0.28)			Worse vs. improved	−0.41 (−0.76 to −0.06)	0.016
Improved	0.47 (0.28 to 0.67)	0.21 (−0.06 to 0.47)			No change vs. improved	−0.16 (−0.41 to 0.09)	0.28
Sleep duration							
Decreased	0.11 (−0.03 to 0.25)	0.01 (−0.23 to 0.26)	1.7 (2)	0.19	Decrease vs. no change	−0.01 (−0.25 to 0.22)	0.99
No change	0.21 (0.04 to 0.38)	0.03 (−0.21 to 0.27)			Decrease vs. increase	−0.17 (−0.42 to 0.07)	0.24
Increased	0.27 (0.12 to 0.42)	0.19 (−0.06 to 0.43)			No change vs. increase	−0.16 (−0.40 to 0.08)	0.26
Sleep medication use							
Increased	−0.03 (−0.48 to 0.43)	−0.44 (−0.83 to −0.052)	4.8 (2)	0.0082	Increase vs. no change	−0.54 (−0.94 to −0.13)	0.0055
No change	0.20 (0.11 to 0.29)	0.10 (−0.12 to 0.31)			Increase vs. decrease	−0.48 (−1.01 to 0.05)	0.084
Decreased	0.32 (−0.09 to 0.73)	0.04 (−0.34 to 0.42)			No change vs. decrease	0.06 (−0.32 to 0.43)	0.94

CI, confidence interval; DF, degrees of freedom; WAS, Work Ability Score.

*Note*: Mean changes (non‐adjusted and adjusted) with 95% CIs in WAS years 2014–2015 according to changes in sleep parameters. Adjusted mean values, *F* values, DF and *p* values are from three different models for multiway analysis of covariance to explain factors affecting the change in work ability. Change in sleep quality, sleep duration and sleep medication use were all entered in different models with WAS change. All models were adjusted for age, gender, body mass index, educational level, disease burden, and WAS level at baseline. Group comparisons between different classes of sleep parameters are also presented. Mean differences in WAS between groups are presented with 95% CIs. The *p* values and 95% CIs for group comparisons are adjusted with Tukey's method.

Disease burden was a significant predictor of the outcome (QoL or WAS change) in every model, but no other background variable was significantly associated with the outcome in any of the models. Complete results from all six multivariable models are presented in Tables S2 and S3.

## 4 DISCUSSION

In this follow‐up study among public sector employees, we showed that changes in sleep quality, sleep duration, and sleep medication use were all significantly associated with changes in QoL. Changes in sleep quality and sleep medication use were also associated with changes in work ability. Improved and unchanged sleep quality compared to worse sleep quality, no change in sleep duration compared to a decrease, and no change in sleep medication use compared to increased use were all associated with favourable changes in QoL. Increased use of sleep medication was associated with a decline in work ability, and the change in work ability also differed significantly between improved and worsened sleep quality.

Tang et al. (2017) have studied the effects of changes in sleep parameters for health and well‐being in a large, population‐based longitudinal survey in the UK (UK Household Study). Their sleep questions were the same as in our study, but they used the GHQ‐12 and the SF‐12 mental and physical component scores as outcome variables over a 4‐year period. They found that an increase in sleep duration and sleep quality and a reduction in sleep medication use were associated with better outcomes in health and well‐being. On the other hand, a decrease in sleep duration and sleep quality and an increase in sleep medication use were associated with poorer outcomes. In our study among the active work force, we expanded this previous research by examining the change in the participants’ general QoL and work ability as the outcome measures. In addition to the background variables used in the study of Tang et al., we also considered the participants’ chronic illnesses as a confounder. This proved to be relevant as chronic illnesses were significantly associated with both QoL and work ability change in all six of our adjusted models. In this setting, we found that changes in sleep quality had the strongest association with changes in QoL (*p* < 0.0001), followed by changes in sleep medication use (*p* = 0.0076), and sleep duration (*p* = 0.031). The same order of these predictors’ effects on health and well‐being was also detected by Tang et al. However, in pairwise comparisons, we did not find significant differences between all the groups of declined and improved sleep parameters, as Tang et al. did in their study. Our setting was somewhat different, but also chronic illnesses, which were not controlled for, may have moderated some of the associations in the UK Household Study. A completely novel finding of our study was that changes in sleep medication use and sleep quality were associated with changes in work ability.

In recent years, the importance of good sleep quality to our health and well‐being has been reported in several studies (Hublin et al., 2018; Marques et al., 2017; Tang et al., 2017). In the present study, especially the association between sleep quality and QoL was prominent: the change in EUROHIS‐QOL was more favourable in the groups of improved and unchanged sleep quality compared to the group of worsened sleep quality. The good results in the group of unchanged sleep quality were based on the favourable results of the participants, whose sleep quality remained continuously good or very good. In our study, as well as in previous literature, especially good sleep quality has been found to be persistent, whereas most of the sleep problems are occasional (Hublin et al., 2018). On that basis, we found it rational to handle the unchanged sleep quality as a single group, mostly reflecting the situation in those whose sleep quality remained good. The change in work ability in the group of improved sleep quality differed also significantly from the group of worsened sleep quality. It is noteworthy that all these significant associations were present although the models were adjusted for several relevant confounding factors, e.g., chronic illnesses, age, and BMI. Thus, our results support the essential role of self‐reported sleep quality as a marker of sleep health in terms of general well‐being and work ability.

According to international recommendations, 7–8 h is considered an optimal sleep duration for the adult population (Hirshkowitz et al., 2015) and excessive short and long sleep are both linked to adverse health conditions (Cappuccio et al., 2010a, 2011; Itani et al., 2017; Jike et al., 2018). Unfavourable sleep duration can be caused by many illnesses such as pain, mental disorders, or obstructive sleep apnea, but also by the modern lifestyle with many night‐time responsibilities and amusements that may shorten sleep duration. In several studies, especially too short sleep has been associated with negative outcomes (Itani et al., 2017; Patel & Hu, 2008; Wang et al., 2012) and an increase in sleep duration is associated with favourable consequences (Chaput et al., 2014; Tang et al., 2017). However, changes towards the optimal sleep duration from either too short or too long sleep might result in the most optimal outcomes, which may confound the effects of shortening or lengthening of the sleep duration. In our study, change in QoL was found more favourable in the group with no change in sleep duration compared to the group of decreased sleep duration, but no other significant associations were detected with QoL or work ability change. The most favourable result in the group of “no change” might be because the proportion of participants sleeping 7–8 h/night was highest in that group at the follow‐up.

In our study population, 14%–15% of the participants reported sleep medication use during the past month, which was approximately on the same level (16%) as in the study of Tang et al. (2017), where they used the same question about sleep medications as in our study. Only 7% of the participants in the group of unchanged sleep medication use were using sleep medications in our study, and they had higher mean scores in both EUROHIS‐QOL and WAS at the baseline and at the follow‐up than participants in the groups of increased or decreased use of sleep medications (data not shown). In the adjusted models, both EUROHIS‐QOL and WAS changes were significantly more favourable in the group of unchanged use of sleep medication compared to the group of increased use. Adjusted WAS estimate even declined significantly (−0.44) in the group of increased use, which did not happen in any other group of the sleep parameters. EUROHIS‐QOL and WAS changes in the group of decreased use of sleep medication were more favourable than those with increased use, but the differences between the changes in the outcomes were not significant. However, problems with sleeping that have led to the use of sleep medications at some point seem to be associated with more unfavourable change in QoL and work ability compared to the situation where sleep medication has not been needed.

### 4.1 Strengths and limitations of the study

Our study had some clear strengths. We assessed the change in QoL, work ability and the sleep parameters by analysing repeated measurements rather than the participants’ perceptions of occurred change. Our assessment tools for sleep, QoL and work ability were all self‐reported, which accentuates the value of the participants’ subjective experience about these factors. It is notable that also the diagnosis of insomnia is based on subjective assessment of sleep rather than objective measurements (ICD 10, 2019). The participants completed the questionnaires at home before the study visits, and weight and height were measured by trained medical staff. Information about chronic diseases was collected both with questionnaires and from medical records, which improves the accuracy of the data. All the participants were in the public sector active work force and had a constant employment status during the follow‐up. The results are thus controlled in terms of employment status.

A major limitation of the study is that despite its longitudinal nature, we cannot report any causality, as changes in sleep parameters and outcomes have been measured concurrently. In addition, the stable employment status of the active work force among all participants may have led to a possible healthy worker effect (Li & Sung, 1999). Furthermore, the initial response rate in the first part of the study in 2014 was only 32.5% (and 85% of them attended the study in 2015). It is known that response rates in email surveys tend to be lower than in mail surveys (Shih & Fan, 2009), but it can nevertheless cause selection bias. It is possible that the healthiest sub‐group of the work force is also the most willing to attend voluntary health surveys, which may result in the possibility that our findings reflect the situation in the mainly healthy section of the work force and, subsequently, may reduce the generalisability of the results. However, the mean annual rate of sickness absence days did not vary significantly between the study participants and the non‐participants in the included employment sectors, as described earlier (Bergman et al., 2020). The assessment of sleep in this study was made with three individual self‐reported items derived from the PSQI, and we did not have any objective measurement of sleep. According to current understanding, simple self‐reported sleep duration is not a validated or reliable measure of biological sleep duration (Bianchi et al., 2017). Nevertheless, also in a meta‐analysis of short sleep duration and health outcomes, most of the included studies had assessed sleep duration with self‐reported tools, and the writers of the meta‐analysis even preferred the self‐reported data over objective measurements because of their better applicability in general community settings (Itani et al., 2017). Furthermore, we unfortunately do not have data about possible menopausal symptoms, which may have affected our results in women.

## 5 CONCLUSIONS

The main findings of the present study were that an improvement in self‐reported sleep quality was very significantly associated with a positive change in QoL, and increased use of sleep medication was associated with a decline in work ability during a 1‐year follow‐up among public sector employees. Changes in sleep medication use and sleep duration were also associated with changes in QoL and changes in sleep quality with changes in work ability. All these associations were present in models adjusted for several potential confounding factors, including chronic illnesses.

These findings accentuate the understanding that sleep health is an essential element of the well‐being and work ability of employees and should be evaluated routinely at occupational and primary healthcare appointments. Special attention should be paid to patients with a need for sleep medication, and our findings should also encourage employers to invest in programmes aiming for better employee sleep health.

## CONFLICT OF INTEREST

None of the authors have any conflicts of interest to declare.

## AUTHOR CONTRIBUTIONS

Elina Bergman, Eliisa Löyttyniemi, Saana Myllyntausta, Päivi Rautava and Päivi E. Korhonen contributed to the conception or design of the work, and to the acquisition, analysis or interpretation of data for the work. Elina Bergman and Päivi E. Korhonen drafted the manuscript. All authors critically revised the manuscript, gave final approval, and agreed to be accountable for all aspects of work, ensuring integrity and accuracy.

## Supporting information


**Table S1** Characteristics of the study population with a comparison to participants excluded from the analyses.

**Table S2** Model‐based estimates with 95% confidence intervals (CIs), *F* values, degrees of freedom (DF) and *p* values (*p*) from three models for multiway analysis of covariance to explain factors affecting EUROHIS‐QOL change.

**Table S3** Model‐based estimates with 95% confidence intervals (CIs), *F* values, degrees of freedom (DF) and *p* values (*p*) from three models for multiway analysis of covariance to explain factors affecting Work Ability Score (WAS) change.

## Data Availability

The data that support the findings of this study are available from the corresponding author upon reasonable request.
