# Supplementary material for: Temporal changes in self‐reported sleep quality, sleep duration and sleep medication use in relation to temporal changes in quality of life and work ability over a 1‐year period among Finnish municipal employees
Source: J Sleep Res. 2022 Apr 15;31(6):e13605. doi: 10.1111/jsr.13605 (PMC9787037; doi:10.1111/jsr.13605)
Supplement: Supplementary file 1 — Table S1 Characteristics of the study population with a comparison to participants excluded from the analyses. [file JSR-31-e13605-s003.docx]

Table S1. Characteristics of the study population with a comparison to participants excluded from the analyses.

|  | | n total [n study population / n drop-outs] | Study population | Drop-outs | p |
| --- | --- | --- | --- | --- | --- |
| Gender n (%) | | 829 [637/192] |  |  | 0.33 |
|  | Female |  | 561 (88.1) | 164 (85.4) |  |
|  | Male |  | 76 (11.9) | 28 (14.58) |  |
| Age n (%) | | 829 [637/192] |  |  | 0.036 |
|  | <45 years |  | 212 (33.3) | 58 (30.2) |  |
|  | 45–55 years |  | 235 (36.9) | 58 (30.2) |  |
|  | >55 years |  | 190 (29.8) | 76 (39.6) |  |
| Age mean, years (SD) | | 829 [637/192] | 48.0 (9.5) | 49.4 (9.9) | 0.075 |
| Body mass index n (%) | | 697 [637/60] |  |  | 0.79 |
|  | <30 kg/m² |  | 498 (78.2) | 46 (76.7) |  |
|  | ≥30 kg/m² |  | 139 (21.8) | 14 (23.3) |  |
| Vocational education n (%) | | 816 [637/179] |  |  | 0.010 |
|  | Vocational school |  | 16 (2.5) | 10 (5.6) |  |
|  | College level |  | 322 (50.6) | 104 (58.1) |  |
|  | University level |  | 299 (46.9) | 65 (36.3) |  |
| Disease burden 2014 n (%) | | 829 [637/192] |  |  | 1.00 |
|  | Yes |  | 383 (60.1) | 116 (60.4) |  |
|  | No |  | 254 (39.9) | 76 (39.6) |  |
| Sleep quality 2014 n (%) | | 816 [632/184] |  |  | 0.63 |
|  | Very good |  | 71 (11.2) | 27 (14.7) |  |
|  | Good |  | 385 (60.9) | 106 (57.6) |  |
|  | Poor |  | 161 (25.5) | 46 (25.0) |  |
|  | Very Poor |  | 15 (2.4) | 5 (2.7) |  |
| Sleep quality 2015 n (%) | | 703 [635/68] |  |  | 0.47 |
|  | Very good |  | 95 (15.0) | 8 (11.7) |  |
|  | Good |  | 397 (62.5) | 39 (57.4) |  |
|  | Poor |  | 130 (20.5) | 19 (27.9) |  |
|  | Very Poor |  | 13 (2.1) | 2 (2.9) |  |
| Sleep quality change n (%) | | 693 [630/63] |  |  | 0.51 |
|  | Worse |  | 87 (13.8) | 10 (15.87) |  |
|  | No change |  | 404 (64.1) | 43 (68.25) |  |
|  | Improved |  | 139 (22.1) | 10 (15.87) |  |
| Sleep duration change n (%) | | 663 [637/26] |  |  | 0.50 |
|  | Decreased |  | 196 (30.8) | 7 (26.9) |  |
|  | No change |  | 248 (38.9) | 8 (30.8) |  |
|  | Increased |  | 193 (30.3) | 11 (42.3) |  |
| Sleep medication use 2014 n (%) | | 816 [633/183] |  |  | 0.46 |
|  | No |  | 539 (85.2) | 160 (87.4) |  |
|  | <1 night /week |  | 43 (6.8) | 10 (5.5) |  |
|  | 1–2 nights /week |  | 23 (3.6) | 3 (1.6) |  |
|  | ≥ 3 nights /week |  | 28 (4.4) | 10 (5.5) |  |
| Sleep medication use 2015 n (%) | | 703 [634/69] |  |  | 0.0040 |
|  | No |  | 547 (86.3) | 51 (73.9) |  |
|  | <1 night /week |  | 44 (6.9) | 5 (7.3) |  |
|  | 1–2 nights /week |  | 14 (2.2) | 3 (4.4) |  |
|  | ≥ 3 nights /week |  | 29 (4.6) | 10 (14.5) |  |
| Sleep medication change n (%) | | 693 [630/63] |  |  | 0.0063 |
|  | Increased |  | 40 (6.4) | 10 (15.9) |  |
|  | No change |  | 543 (86.2) | 52 (82.5) |  |
|  | Decreased |  | 47 (7.5) | 1 (1.6) |  |
| EuroHIS mean 2014 | | 822 [637/185] | 3.94 (3.90 to 3.98) | 3.87 (3.79 to 3.96) | 0.15 |
| EuroHIS mean 2015 | | 706 [637/69] | 4.08 (4.04 to 4.12) | 4.00 (3.87 to 4.13) | 0.23 |
| EuroHIS change | | 701 [637/64] | 0.14 (0.11 to 0.17) | 0.08 (-0.02 to 0.18) | 0.20 |
| WAS mean 2014 | | 820 [637/183] | 8.16 (8.06 to 8.26) | 8.16 (7.96 to 8.37) | 0.98 |
| WAS mean 2015 | | 703 [637/66] | 8.36 (8.26 to 8.46) | 8.03 (7.62 to 8.44) | 0.057 |
| WAS change | | 696 [637/59] | 0.20 (0.11 to 0.29) | -0.20 (-0.66 to 0.26) | 0.015 |

Information about gender, age, body mass index, and vocational education level at baseline (2014). Difference between the study population and all the participants was evaluated using a chi-square test or a one-way analysis of variance (ANOVA). P-Value (p) is for the difference between the groups.
