# Supplementary material for: Temporal changes in self‐reported sleep quality, sleep duration and sleep medication use in relation to temporal changes in quality of life and work ability over a 1‐year period among Finnish municipal employees
Source: J Sleep Res. 2022 Apr 15;31(6):e13605. doi: 10.1111/jsr.13605 (PMC9787037; doi:10.1111/jsr.13605)
Supplement: Supplementary file 2 — Table S2 Model‐based estimates with 95% confidence intervals (CIs), F values, degrees of freedom (DF) and p values (p) from three models for multiway analysis of covariance to explain factors affecting EUROHIS‐QOL change. [file JSR-31-e13605-s002.docx]

Table S2. Model-based estimates with 95% confidence intervals (CI), F-values, Degrees of freedom (DF) and p-values (p) from three models for multiway analysis of covariance to explain factors affecting EUROHIS-QOL change.

|  | | Model for EUROHIS-QOL change with sleep quality change | | | Model for EUROHIS-QOL change with sleep duration change | | | Model for EUROHIS-QOL change with sleep medication change | | |
| --- | --- | --- | --- | --- | --- | --- | --- | --- | --- | --- |
|  |  | Mean change adjusted estimate (95% CI) | F value (DF) | p | Mean change adjusted estimate (95% CI) | F value (DF) | p | Mean change adjusted estimate (95% CI) | F value (DF) | p |
| Gender | |  | 1.13 (1) | 0.29 |  | 0.82 (1) | 0.36 |  | 0.76 (1) | 0.38 |
|  | Female | 0.05 (-0.02 to 0.11) |  |  | 0.08 (0.02 to 0.15) |  |  | 0.02 (-0.06 to 0.10) |  |  |
|  | Male | 0.09 (-0.01 to 0.19) |  |  | 0.12 (0.03 to 0.22) |  |  | 0.06 (0.05 to 0.17) |  |  |
| Age | |  | 0.04 (2) | 0.96 |  | 0.10 (2) | 0.91 |  | 0.33 (2) | 0.72 |
|  | <45 years | 0.07 (-0.02 to 0.16) |  |  | 0.09 (0.01 to 0.18) |  |  | 0.02 (-0.80 to 0.12) |  |  |
|  | 45–55 years | 0.07 (-0.01 to 0.15) |  |  | 0.11 (0.03 to 0.18) |  |  | 0.05 (-0.04 to 0.14) |  |  |
|  | >55 years | 0.06 (-0.02 to 0.15) |  |  | 0.11 (0.03 to 0.19) |  |  | 0.04 (-0.05 to 0.14) |  |  |
| BMI | |  | 1.53 (1) | 0.22 |  | 1.27 (1) | 0.26 |  | 1.24 (1) | 0.27 |
|  | <30 kg/m² | 0.09 (0.02 to 0.16) |  |  | 0.12 (0.05 to 0.19) |  |  | 0.06 (-0.03 to 0.14) |  |  |
|  | ≥30 kg/m² | 0.05 (-0.04 to 0.14) |  |  | 0.08 (0.00 to 0.19) |  |  | 0.02 (-0.08 to 0.12) |  |  |
| Vocational education | |  | 2.78 (2) | 0.063 |  | 1.91 (2) | 0.15 |  | 2.15 (2) | 0.12 |
|  | Vocational school | -0.07 (-0.25 to 0.11) |  |  | 0.00 (-0.18 to 0.17) |  |  | -0.08 (-0.27 to 0.11) |  |  |
|  | College level | 0.14 (0.09 to 0.20) |  |  | 0.17 (0.11 to 0.22) |  |  | 0.11 (0.04 to 0.18) |  |  |
|  | University level | 0.13 (0.07 to 0.18) |  |  | 0.14 (0.09 to 0.20) |  |  | 0.08 (0.01 to 0.15) |  |  |
| Disease burden | |  | 6.35 (1) | 0.012 |  | 7.49 (1) | 0.0064 |  | 8.29 (1) | 0.0041 |
|  | No | 0.10 (0.02 to 0.19) |  |  | 0.14 (0.06 to 0.22) |  |  | 0.08 (-0.01 to 0.17) |  |  |
|  | Yes | 0.03 (-0.04 to 0.11) |  |  | 0.06 (-0.01 to 0.14) |  |  | 0.00 (-0.09 to 0.08) |  |  |
| Sleep quality | |  | 9.89 (2) | <0.0001 |  |  |  |  |  |  |
|  | Worse | -0.05 (-0.15 to 0.05) |  |  |  |  |  |  |  |  |
|  | No change | 0.10 (0.03 to 0.18) |  |  |  |  |  |  |  |  |
|  | Improved | 0.16 (0.07 to 0.24) |  |  |  |  |  |  |  |  |
| Sleep duration | |  |  |  |  | 3.50 (2) | 0.031 |  |  |  |
|  | Decreased |  |  |  | 0.05 (-0.03 to 0.13) |  |  |  |  |  |
|  | No change |  |  |  | 0.13 (0.05 to 0.21) |  |  |  |  |  |
|  | Increased |  |  |  | 0.13 (0.05 to 0.21) |  |  |  |  |  |
| Sleep medication use | |  |  |  |  |  |  |  | 4.92 (2) | 0.0076 |
|  | Increased |  |  |  |  |  |  | -0.06(-0.19 to 0.07) |  |  |
|  | No change |  |  |  |  |  |  | 0.12 (0.04 to 0.19) |  |  |
|  | Decreased |  |  |  |  |  |  | 0.06 (-0.07 to 0.18) |  |  |

Change in sleep quality, sleep duration and sleep medication use were all entered in different models with same background variables and EUROHIS-QOL level at baseline. P-values indicate if the variable is significantly associated with the outcome in the model.

CI, Confidence interval; DF, Degrees of freedom; EUROHIS-QOL, EUROHIS-QOL 8-item index
