# Supplementary material for: Temporal changes in self‐reported sleep quality, sleep duration and sleep medication use in relation to temporal changes in quality of life and work ability over a 1‐year period among Finnish municipal employees
Source: J Sleep Res. 2022 Apr 15;31(6):e13605. doi: 10.1111/jsr.13605 (PMC9787037; doi:10.1111/jsr.13605)
Supplement: Supplementary file 3 — Table S3 Model‐based estimates with 95% confidence intervals (CIs), F values, degrees of freedom (DF) and p values (p) from three models for multiway analysis of covariance to explain factors affecting Work Ability Score (WAS) change. [file JSR-31-e13605-s001.docx]

Table S3. Model-based estimates with 95% confidence intervals (CI), F-values, Degrees of freedom (DF) and p-values (p) from three models for multiway analysis of covariance to explain factors affecting Work Ability Score (WAS) change.

|  | | Model for WAS change with sleep quality change | | | Model for WAS change with sleep duration change | | | Model for WAS change with sleep medication change | | |
| --- | --- | --- | --- | --- | --- | --- | --- | --- | --- | --- |
|  |  | Mean change adjusted estimate (95% CI) | F value (DF) | p | Mean change adjusted estimate (95% CI) | F value (DF) | p | Mean change adjusted estimate (95% CI) | F value (DF) | p |
| Gender | |  | 1.23 (1) | 0.27 |  | 1.57 (1) | 0.21 |  | 1.97 (1) | 0.16 |
|  | Female | 0.09 (-0.12 to 0.30) |  |  | 0.16 (-0.04 to 0.36) |  |  | -0.01 (-0.25 to 0.23) |  |  |
|  | Male | -0.06 (-0.36 to 0.25) |  |  | 0.00 (-0.30 to 0.29) |  |  | -0.19 (-0.52 to 0.14) |  |  |
| Age | |  | 1.11 (2) | 0.33 |  | 1.15 (2) | 0.32 |  | 1.14 (2) | 0.32 |
|  | <45 years | 0.07 (-0.20 to 0.34) |  |  | 0.12 (-0.14 to 0.38) |  |  | -0.07 (-0.36 to 0.24) |  |  |
|  | 45–55 years | 0.05 (-0.19 to 0.30) |  |  | 0.13 (-0.10 to 0.36) |  |  | -0.05 (-0.32 to 0.23) |  |  |
|  | >55 years | -0.08 (-0.33 to 0.18) |  |  | -0.01 (-0.25 to 0.22) |  |  | -0.19 (-0.47 to 0.09) |  |  |
| BMI | |  | 0.01 (1) | 0.94 |  | 0.05 (1) | 0.83 |  | 0.18 (1) | 0.67 |
|  | <30 kg/m² | 0.01 (-0.21 to 0.24) |  |  | 0.07 (-0.14 to 0.28) |  |  | -0.12 (-0.38 to 0.13) |  |  |
|  | ≥30 kg/m² | 0.02 (-0.25 to 0.29) |  |  | 0.09 (-0.17 to 0.35) |  |  | -0.08 (-0.38 to 0.22) |  |  |
| Vocational education | |  | 1.15 (2) | 0.32 |  | 0.81 (2) | 0.45 |  | 0.79 (2) | 0.45 |
|  | Vocational school | -0.24 (-0.79 to 0.31) |  |  | -0.13 (-0.66 to 0.40) |  |  | -0.33 (-0.89 to 0.24) |  |  |
|  | College level | 0.12 (-0.06 to 0.29) |  |  | 0.15 (-0.01 to 0.32) |  |  | 0.00 (-0.21 to 0.21) |  |  |
|  | University level | 0.17 (0.00 to 0.35) |  |  | 0.21 (0.04 to 0.37) |  |  | 0.02 (-0.19 to 0.24) |  |  |
| Disease burden | |  | 7.19 (1) | 0.0075 |  | 8.09 (1) | 0.0046 |  | 8.75 (1) | 0.0032 |
|  | No | 0.13 (-0.12 to 0.39) |  |  | 0.20 (-0.04 to 0.44) |  |  | 0.03 (-0.25 to 0.31) |  |  |
|  | Yes | -0.10 (-0.33 to 0.13) |  |  | -0.05 (-0.27 to 0.17) |  |  | -0.23 (-0.50 to 0.03) |  |  |
| Sleep quality | |  | 3.84 (2) | 0.022 |  |  |  |  |  |  |
|  | Worse | -0.20 (-0.51 to 0.10) |  |  |  |  |  |  |  |  |
|  | No change | 0.05 (-0.18 to 0.28) |  |  |  |  |  |  |  |  |
|  | Improved | 0.21 (-0.06 to 0.47) |  |  |  |  |  |  |  |  |
| Sleep duration | |  |  |  |  | 1.67 (2) | 0.19 |  |  |  |
|  | Decreased |  |  |  | 0.01 (-0.23 to 0.26) |  |  |  |  |  |
|  | No change |  |  |  | 0.03 (-0.21 to 0.27) |  |  |  |  |  |
|  | Increased |  |  |  | 0.19 (-0.06 to 0.43) |  |  |  |  |  |
| Sleep medication use | |  |  |  |  |  |  |  | 4.84 (2) | 0.0082 |
|  | Increased |  |  |  |  |  |  | -0.44 (-0.83 to -0.05) |  |  |
|  | No change |  |  |  |  |  |  | 0.10 (-0.12 to 0.31) |  |  |
|  | Decreased |  |  |  |  |  |  | 0.04 (-0.34 to 0.42) |  |  |

Change in sleep quality, sleep duration and sleep medication use were all entered in different models with same background variables and WAS level at baseline. P-values indicate if the variable is significantly associated with the outcome in the model.

CI, Confidence interval; DF, Degrees of freedom; WAS, Work Ability Score
